# Determination of Nitric Oxide and Its Metabolites in Biological Tissues Using Ozone-Based Chemiluminescence Detection: A State-of-the-Art Review

**DOI:** 10.3390/antiox13020179

**Published:** 2024-01-31

**Authors:** Junjie Li, Anthea LoBue, Sophia K. Heuser, Miriam M. Cortese-Krott

**Affiliations:** 1 Myocardial Infarction Research Laboratory, Department of Cardiology, Pulmonology, and Angiology, Medical Faculty, Heinrich-Heine-University, 40225 Düsseldorf, Germany; junjie.li@med.uni-duesseldorf.de (J.L.); anthea.lobue@med.uni-duesseldorf.de (A.L.); sophiakatharina.heuser@med.uni-duesseldorf.de (S.K.H.); 2 CARID, Cardiovascular Research Institute Düsseldorf, Medical Faculty, Heinrich-Heine-University, 40225 Düsseldorf, Germany; 3 Department of Physiology and Pharmacology, Karolinska Institute, 17177 Stockholm, Sweden

**Keywords:** chemiluminescence detection, ^•^NO metabolites, vascular function, clinical studies

## Abstract

Ozone-based chemiluminescence detection (CLD) has been widely applied for determining nitric oxide (^•^NO) and its derived species in many different fields, such as environmental monitoring and biomedical research. In humans and animals, CLD has been applied to determine exhaled ^•^NO and ^•^NO metabolites in plasma and tissues. The main advantages of CLD are high sensitivity and selectivity for quantitative analysis in a wide dynamic range. Combining CLD with analytical separation techniques like chromatography allows for the analytes to be quantified with less disturbance from matrix components or impurities. Sampling techniques like microdialysis and flow injection analysis may be coupled to CLD with the possibility of real-time monitoring of ^•^NO. However, details and precautions in experimental practice need to be addressed and clarified to avoid wrong estimations. Therefore, using CLD as a detection tool requires a deep understanding of the sample preparation procedure and chemical reactions used for liberating ^•^NO from its derived species. In this review, we discuss the advantages and pitfalls of CLD for determining ^•^NO species, list the different applications and combinations with other analytical techniques, and provide general practical notes for sample preparation. These guidelines are designed to assist researchers in comprehending CLD data and in selecting the most appropriate method for measuring ^•^NO species.

## 1. Introduction

**Figure 1:**
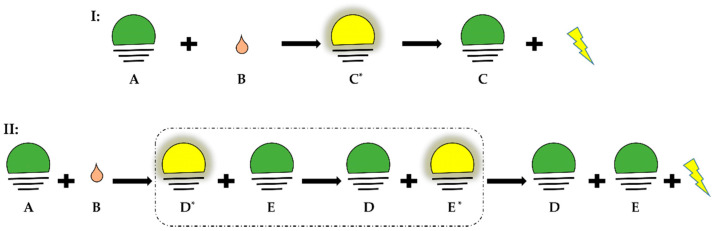
Direct and indirect chemiluminescence. Green: basal state; yellow: excited state. (**I**) Direct chemiluminescence, A: chemiluminescent molecule, B: oxidant, C*: excited state of intermediate, C: ground state of intermediate. (**II**) Indirect chemiluminescence, D*: excited state of intermediate, D: ground state of intermediate, E: ground state of fluorophore, E*: excited state of fluorophore.

Chemiluminescence is defined as the emission of light as the result of a chemical reaction. The reactants or intermediates are chemically activated via oxidation into an electronically excited state and then can release light by two distinct mechanisms defined as direct and indirect chemiluminescence (Figure 1). In direct chemiluminescence, the chemiluminescent molecules (A) are oxidized to unstable excited intermediates (C*) that then return to the ground state (C) by releasing energy in the form of photons. In indirect chemiluminescence, the excited intermediates (D*), instead of directly decaying, transfer energy through an optical process to the surrounding fluorophores (E), which then become excited (E*) and release energy by light emission. This phenomenon is called chemiluminescence resonance energy transfer of light.

Luminol was one of the first chemiluminescent molecules applied for the detection of oxidants and free radicals using direct chemiluminescence [1,2]. Luminol emits blue light with a wavelength of 425 nm after being oxidized [1,2,3,4]. Luminol chemiluminescence was applied for the detection of oxidants such as superoxide, hydrogen peroxide, and peroxynitrite [1,5,6]. However, the use of luminol chemiluminescence has strong limitations and is only semiquantitative. The main reason is that the oxidation of luminol is an autocatalytic process, which yields unspecific signal potentiation. Moreover, the oxidation yield is enhanced by the presence of bicarbonate–carbon dioxide (through the formation of ONOOCO_2_^−^ that decomposes into CO_3_^•−^). The oxidation can be inhibited by free thiols and other antioxidants [7]. Therefore, luminol-dependent chemiluminescence assay is now only applied to analyze overall oxidant formation under specific and controlled conditions [1,8].

Ozone-based chemiluminescence detection (CLD) for nitric oxide (^•^NO) species is well recognized as a highly sensitive and specific approach for quantifying gaseous ^•^NO. The detector quantifies the light produced by the reaction of ^•^NO with ozone in the gas phase [9]. The reaction produces excited nitrogen dioxide (NO_2_^*^) (Reaction (1)), which then emits photons when it returns to the ground state (Reaction (2)). The emitted light is then amplified with a photomultiplier tube and detected.

^•^NO + O_3_ → NO_2_^*^ + O_2_ (1)

NO_2_^*^ → NO_2_ + *hv* (2)

**Figure 2:**
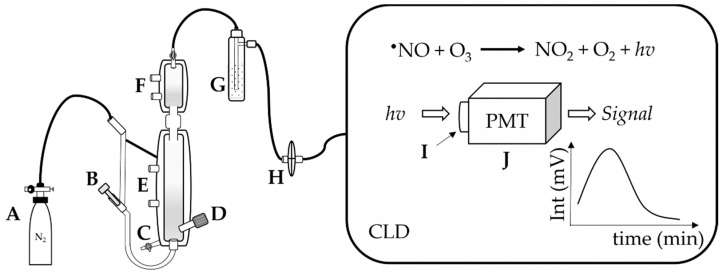
Apparatus for ozone-based chemiluminescence. A: supply gas, N_2_, B: supply gas fine control for purging, C: waste outlet, D: injection port of sample, E: heating circulation, F: cooling circulation, G: NaOH trap, H: gas filter; in the CLD: the emission *hv* is collected by the I: optical filter and then converted by the J: photomultiplier tube (PMT) to amplify the signal in mV.

Typically, ^•^NO is generated in a reaction chamber purged with an inert carrier gas (N_2_ or argon), which carries the generated ^•^NO along the tubing connecting the chamber to the CLD. The chamber contains a reductive or oxidative solution prepared for the reaction under acidic or neutral conditions and temperated with defined temperature conditions (e.g., 60 °C). The settings of the reaction chamber depend on the type of analyte and the type of ^•^NO derivatives that one may want to quantify. After leaving the reaction chamber, the carrier gas together with ^•^NO is then purged into a “NaOH trap”, consisting of a solution of NaOH (1 N), which prevents high-temperature acid vapor from entering and damaging the chemiluminescence detector (Figure 2) [10,11].

## 2. NO and Its Biologically Relevant Derivatives

^•^NO is a moderately reactive uncharged free radical, which attracted a lot of attention in the late 1980s for its biological properties. It was found to be the endothelium-derived relaxing factor (EDRF), which uncovered how endothelial cells are involved in the vasodilatory effect of smooth muscle cells in the vasculature [11,12,13]. The nature of EDRF as ^•^NO was indeed revealed using CLD [11,14]. The Nobel Prize in Physiology or Medicine for 1998 was awarded jointly to Robert F. Furchgott, Louis J. Ignarro, and Ferid Murad for their discoveries concerning “nitric oxide as a signaling molecule in the cardiovascular system”.

**Figure 3:**
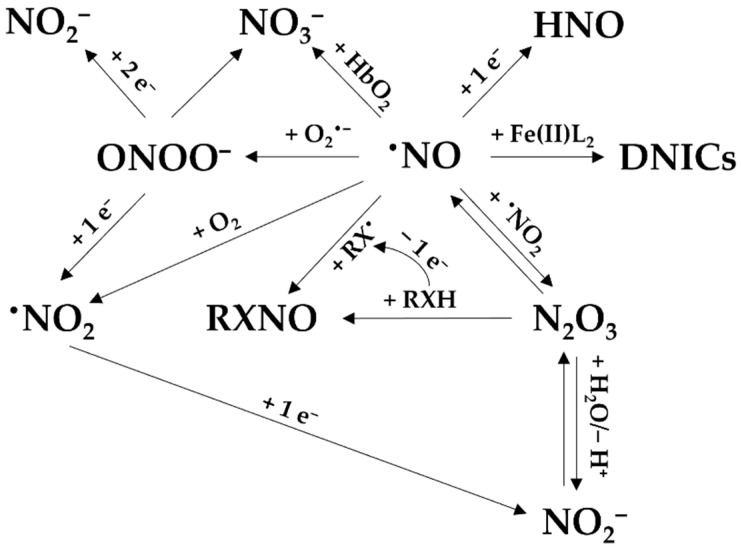
The reactions of ^•^NO to form its derived species. Nitric oxide (^•^NO) can react with O_2_^•−^ to form peroxynitrite (ONOO−), which then can yield nitrate (NO_3_^−^) or be reduced to NO_2_^−^ and ^•^NO_2_. NO_3_^−^ can be also derived from the reaction between ^•^NO and oxyhemoglobin (HbO_2_). Nitrosyl (HNO) can be formed by reduction of ^•^NO. ^•^NO can bind to iron (Fe(II)) to form dinitrosyl iron complexes (DNICs). ^•^NO can also react with nitrogen dioxide (^•^NO_2_), with N_2_O_3_ as intermediate, to generate nitrite (NO_2_^−^) and nitrosated product (RXNO).

By reacting with other radicals and molecules produced in a biological environment, ^•^NO leads to the formation of reactive nitrogen species. The reactive nitrogen species are in turn involved in the oxidation or nitrosation of biomolecules [15,16]. Other downstream species include dinitrogen trioxide (N_2_O_3_), nitrosothiols and nitrosamines (often abbreviated as RSNO and RNNO, respectively), intracellular dinitrosyl iron complexes (DNICs), nitroxyl (HNO), ^•^NO_2_, peroxynitrite (ONOO^−^), nitrite, and nitrate (Figure 3) [8]. Together with reactive oxygen and sulfur species, they are part of the *reactive species interactome* [17].

N_2_O_3_, as an intermediate in the autoxidation of ^•^NO, is derived from the reaction of ^•^NO with ^•^NO_2_ (Reactions (3) and (4)). N_2_O_3_ can be then hydrolyzed to two molecules of nitrite or rapidly nitrosate thiols and amines, leading to the formation of RSNO, RNNO, and nitrite (Reactions (5) and (6)) [16,18,19,20,21].

2^•^NO + O_2_ → 2^•^NO_2_ (3)

^•^NO + ^•^NO_2_ ⇌ N_2_O_3_ (4)

N_2_O_3_ + H_2_O → 2HNO_2_ ⇌ 2NO_2_^−^ + 2H^+^ (5)

N_2_O_3_ + R_2_NH → R_2_NNO + NO_2_^−^ + H^+^ (6)

Instead of direct nitrosation of thiols by N_2_O_3_, RSNO was also proposed to be formed in the reaction of RS^−^ with ^•^NO_2_ to generate RS^•^, which then reacts with ^•^NO to obtain RSNO (Reactions (7) and (8)) [22].

RS^−^ + ^•^NO_2_ → RS^•^ + NO_2_^−^ (7)

RS^•^ + ^•^NO → RSNO (8)

In addition, ^•^NO can target also Fe-heme and bind to iron from or partially from the chelatable iron pool together with ligands such as glutathione to form DNICs [23].

NO^−^ is formed by the one-electron reduction of ^•^NO, which is found only in its protonated form, HNO [8]. HNO could also react with oxygen leading to the formation of ONOO^−^. However, this reaction has low relevance under biological conditions due to a relatively slow reaction rate (Reaction (9)) [24,25].

NO^−^ + O_2_ → OONO^−^ (9)

ONOO^−^ is formed mainly by the reaction between ^•^NO and O_2_^•−^ (Reaction (10)). ONOO^−^ can undergo one- or two-electron reduction yielding ^•^NO_2_ and NO_2_^−^, respectively [26,27]. ONOO^−^ can be also isomerized to NO_3_^−^ in the presence of metmyoglobin or methemoglobin as a catalyst (Reaction (11)) [28,29].

^•^NO + O_2_^•−^ → OONO^−^ (10)

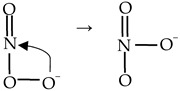
(11)

Nitrate can be derived by the reaction between ^•^NO and oxyhemoglobin (Reaction (12)) [30]. Under certain conditions such as in the presence of oral bacteria or the enzyme xanthine oxidoreductase, nitrate can be reduced back to nitrite. Nitrite is an important source of ^•^NO in the body [31,32,33,34,35,36].

Nitrite can be reduced to ^•^NO by proteins such as deoxyhemoglobin, deoxymyoglobin, and other globins under hypoxic conditions. These mechanisms contribute to many biological processes such as vasodilation, neurotransmission, immune response, and other physiological signaling pathways (Reaction (13)) [37,38].

^•^NO + oxyHb → NO_3_^−^ + metHb (12)

NO_2_^−^ + deoxyHb → ^•^NO + metHb (13)

^•^NO was also described to react with deoxyhemoglobin to form nitrosylhemoglobin, where ^•^NO forms a complex with the iron heme. Nitrosylhemoglobin was described as a transporter of ^•^NO bioactivity, as an intermediate for the formation of s-nitrosohemoglobin, and as an intermediate formed during nitrite reduction into ^•^NO by deoxyhemoglobin [37,39,40,41].

## 3. Measurement of ^•^NO Metabolites Using Chemiluminescence

In the reaction chamber, the ^•^NO metabolites need to be converted back to ^•^NO by reduction (nitrate, nitrite, RSNO, RNNO) or oxidation reactions (NO-heme or DNIC).

### 3.1. Reduction of Nitrite, RSNO, and RNNO with Tri-Iodide, Cys/CuCl, and Hydroquinone/Quinone

The most widely used reductive solution applied in CLD is tri-iodide. The tri-iodide-based reductive reagent consists of potassium iodide (KI) and iodine (I_2_) in glacial acetic acid. Under acidic conditions, nitrite can be protonated to form nitrous acid with acid dissociation constant at a logarithmic scale (pK_a_) of 3.4. Nitrous acid is unstable at low pH, for example, in glacial acetic acid, it goes through disproportionation to form ^•^NO or by-product N_2_O_3_, which interferes with the detection by variation in the background signal. The KI/I_2_ mixture is very widely used for reducing nitrite to ^•^NO in biological samples (Reaction (14)) [10,42]. In addition to nitrite, the tri-iodide assay can also be applied for the detection of other biological ^•^NO metabolites such as nitrosated (*S*- or *N*-nitroso) products (RXNO) (Reactions (15)–(17)).

I^−^ + NO_2_^−^ + 2H^+^ → ^•^NO + 1/2 I_2_ + H_2_O (14)

I_2_ + I^−^ → I_3_^−^ (15)

I_3_^−^ + 2RSNO → 3I^−^ + RS-SR + 2NO^+^ (16)

2NO^+^ + 2I^−^ → 2NO + I_2_ (17)

Sample preparation for the detection of *S*-nitrosated products (RSNO) requires a pre-treatment of the samples with a stabilization solution containing N-ethylmaleimide (NEM) that prevents artificial *S*-nitrosation of free thiols. Treatments with NEM have some drawbacks as NEM is often contaminated by nitrite, and the contamination may vary from one lot number to another. It is therefore necessary to check contamination before adding NEM to samples.

In addition, samples need to be treated with sulfanilamide prior detection. This treatment serves to eliminate nitrite by converting it into an undetectable diazonium cation. This procedure ensures that the detection process specifically captures signals related to RSNO and residual species during the tri-iodide assay.

For the detection of RNNO, samples need to be pre-treated with HgCl_2_. This process converts RSNO into NO^+^ and leads to the subsequent formation of NO_2_^−^. Then, the addition of sulfanilamide will remove the formed NO_2_^−^, thus revealing the “remnants” or the RNNOs. Consequently, the combined pre-treatment with HgCl_2_ and sulfanilamide offers an alternative approach to differentiate RSNO and RNNO from other species [43].

The measurement of RSNO can be also carried out using cysteine/CuCl as a reducing matrix (1 mM L-cysteine/0.1 mM CuCl) with a sensitivity of 10 pmol for the RSNO standards or using a hydroquinone/quinone reducing matrix (0.1 M hydroquinone/0.01 M quinone) with quantitative detection down to 10 nM in PBS buffer or rat plasma (see also Table 1) [44,45].

### 3.2. Measurement of Nitrate with Vanadium Chloride

In comparison to nitrite and other species, nitrate is present at a much higher concentration in biological specimens and makes up the major part of total ^•^NO species. To be reduced, it requires a stronger reductive reagent as compared with tri-iodide or an enzymatic reaction catalyzed by a bacterial nitrate reductase. Chemical reduction of nitrate is carried out with vanadium chloride (VCl_3_) prepared in hydrochloric acid (HCl) in a final concentration of 0.1 mol/L of VCl_3_ in 2 mol/L HCl (Reaction (18)) [46,47]. It is worth mentioning that the VCl_3_/HCl reagent will not just reduce nitrate but also all the other metabolites able to be reduced using the tri-iodide method. To obtain the accurate nitrate level, therefore, the results from the tri-iodide assay need to be subtracted from the observed signals in the VCl_3_/HCl method.

2NO_3_^−^ + 3V^3+^ + 2H_2_O → 2^•^NO + 3VO_2_^+^ + 4H^+^ (18)

### 3.3. Measurement of NO-Heme with Ferricyanide

Gladwin et al. proposed an oxidation method based on one-electron oxidation of NO-heme with ferricyanide for the determination of NO-heme, with a sensitivity down to 5 nM in human blood after removing the background signal of nitrite (Reaction (19)) [48]. This method was further modified by Bryan et al., who used 0.05 M instead of 0.2 M ferricyanide in PBS at pH 7.5 to determine NO-heme in rat tissues such as the brain, heart, liver, etc., as well as in the blood components, plasma, and erythrocytes [20].

NO-heme (Fe^2+^) + Ferricyanide (Fe^3+^) → medHb (Fe^3+^) + Ferrocyanide (Fe^2+^) + ^•^NO (19)

Some common reductive and oxidative solutions for the detection of ^•^NO metabolites are listed in Table 1.

**Table 1:** Reductive and oxidative reaction solution for NO metabolites.

Reaction Solution	Conditions	Target NO Metabolites	References
Iodine/iodide	60 mM I^−^/6 to 20 mM I_2_/1M HCl, RT	NO_2_^−^, RNNO, RSNO; RNNO, RSNO with the addition of sulfanilamide and HgCl_2_	[44]
	56 mM I^−^/2 mM I_2_, 4 mM CuCl, CH_3_COOH, 68 °C		[49]
	45 mM I^−^/10 mM I_2_, CH_3_COOH, 60 °C		[10]
Cysteine/CuCl	1 mM L-cysteine/0.1 mM CuCl	RSNO	[45]
Hydroquinone/quinone	0.1 M hydroquinone/0.01 M quinone	RSNO	[44]
VCl_3_/H^+^	0.1 M vanadium(III) in 2 M HCl	NO_3_^−^, NO_2_^−^, RNNO, RSNO	[50]
Ferricyanide	0.2 M ferricyanide in PBS, pH 7.5	NO-heme	[48]
	0.05 M ferricyanide in PBS, pH 7.5		[20]

## 4. Multi-Level Analytical Approaches for a Comprehensive Analysis of ^•^NO Metabolites

### 4.1. Chemiluminescence Coupled with Chromatography or Mass Spectrometry (MS)

Chemiluminescence compared with other optical techniques such as fluorescence detection, needs no external light source, has a low background signal and, therefore, has a high signal-to-noise ratio and thus is highly sensitive [51,52]. A chemiluminescence nitrogen detector was developed in 1970 based on the reaction of ^•^NO with ozone for continuously monitoring gaseous pollutants in the air [9].

Coupling CLD with analytical separative techniques like gas chromatography (GC) or high-performance liquid chromatography (HPLC) allows for the combination of such a highly sensitive detection technique with powerful analytical separation methods. The selective elution of analytes from the chromatography column can help separate the target analytes from unwanted interferants or impurities in the sample matrix, which could improve the accuracy and the sensitivity of CLD even further [53,54].

#### 4.1.1. Gas Chromatography

A chemiluminescence detector for ^•^NO species coupled with GC was applied for the detection of atmospheric ^•^NO, ammonia, amines, and some nitrogen-containing compounds [9,55,56]. To establish a GC-CLD system, particular attention is required for many critical aspects concerning both GC and CLD. Regarding GC, key considerations include the sample injection (split/splitless, which determine the amount of sample entering the GC system), the column (including type of stationary phase, film thickness, column inner diameter, and length of the column), and the GC-CLD interface (involving a transfer line through which analytes travel from GC to the CLD). These elements are essential components for the proper functioning of the system [56].

GC is an analytical technique for the quantitative analysis of volatile compounds. The volatility of the analytes is important for assuring their volatilization and ability to enter the gaseous mobile phase in a way that they can be transported for further separation through the column. For samples with liquid matrices (like wastewater), the injected samples need to be introduced at a proper flow rate for more complete combustion at the injection port of GC, which eliminates the risk of samples with high water content reaching the chemiluminescence detector [56].

To enhance the peak capacity and improve the resolution, CLD was coupled to two-dimensional gas chromatography (GCxGC) with orthogonality for separation between two applied GC columns. This coupling was used for the quantitative analysis of nitrogen-containing compounds in microalgae-based bio-oils, food samples, and urban aerosol samples [57,58,59].

In GC-CLD, GC introduces the chance for further separation in the GC column; however, it also brings the requirement for the volatility of the samples. The drawbacks of this technique are particularly notable when dealing with complex samples, especially biological tissues or cells. The development of sample preparation methods and instrumental parameters might be time-consuming and costly, which requires more careful consideration and more prudent handling. To the best of our knowledge, GCxGC-CLD was never applied to biological specimens, probably because of the complexity of the sample matrix.

#### 4.1.2. Liquid Chromatography

**Figure 4:**
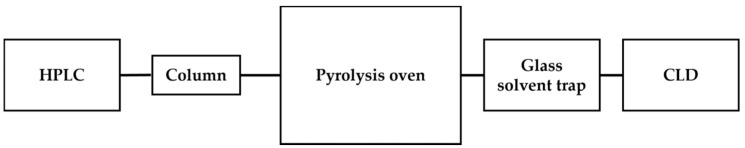
Schematic representation of HPLC-CLD with a pyrolysis oven, as described in Ref. [61]. The eluents from an HPLC column were transformed into the gaseous state within the pyrolysis oven. This system showed some sensitivity issue in the presence of water in aqueous matrixes.

Attempts also have been made to couple CLD with HPLC for detecting ^•^NO species. Using an UPLC system allows for the direct analysis of non-volatile samples in liquid matrices without vaporization [53,60]. HPLC-CLD was applied for the analysis of nitrated polycyclic aromatic hydrocarbons, which are environmental combustion pollutants. In the literature, there is an example of a HPLC-CLD system made by connecting the outlet of an HPLC column to a quartz tubing placed in a pyrolysis oven by using a stainless-steel tubing. The pyrolysis oven is heated at 900 °C and converts the liquid matrices, along with samples, into the gaseous state (Figure 4). It was found that such conversion into the gaseous state before reaching the chemiluminescence detector significantly impacts the sensitivity, which becomes worse with increasing aqueous content in the mobile phase [61].

**Figure 5:**
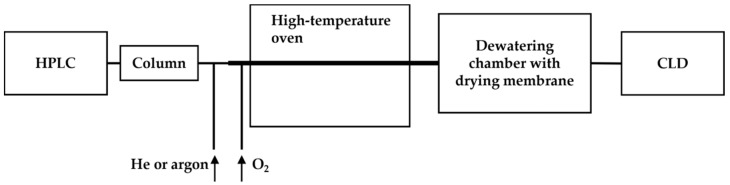
Schematic representation of the HPLC-CLD coupled with a high-temperature oven and a dewatering chamber, as described in Ref. [62]. The dewatering chamber, positioned post the high-temperature oven, serves to remove the aqueous content in the sample flow, thereby enhancing the sensitivity of CLD detection.

To overcome this, a dewatering chamber equipped with a drying membrane was added to the system (as shown in Figure 5). The eluents from the HPLC column were mixed with inert gas (helium or argon) along with oxygen to facilitate the oxidation of all nitrogen-containing compounds to ^•^NO in a high-temperature oven (approximately 1000 °C). The resulting ^•^NO and gases were then transported through a membrane in the dewatering chamber to remove the aqueous content before reacting with ozone in the reaction chamber for chemiluminescence [62]. The modified HPLC-CLD system equipped with a Hamilton PRP-X200 ion chromatography column was validated for the detection limit of ammonium nitrogen down to 5 ng in wastewater and also the capability of profiling nitrate of 80 ng and nitrite of 160 ng in a nitrite–nitrate mixture [62].

The application of HPLC-CLD for the nitrogen-specific detection of commercially synthesized peptides, including crude peptide mixtures, further demonstrated its superiority over simultaneous ultraviolet detection in peptide profiling [53,63]. On the other hand, an HPLC-CLD system was also applied for quantifying nucleosides based on their nitrogen content [60]. Another example of HPLC-CLD system involving a CLD-10A chemiluminescence detector (Shimadzu, Kyoto, Japan) was used for determining hydroxyl-1-nitropyrenes (OHNPs) and metabolites of 1-nitropyrene, including 3-OHNP, 6-OHNP, and 8-OHNP formed by cytochrome P450s [64].

#### 4.1.3. Mass Spectrometry

**Figure 6:**
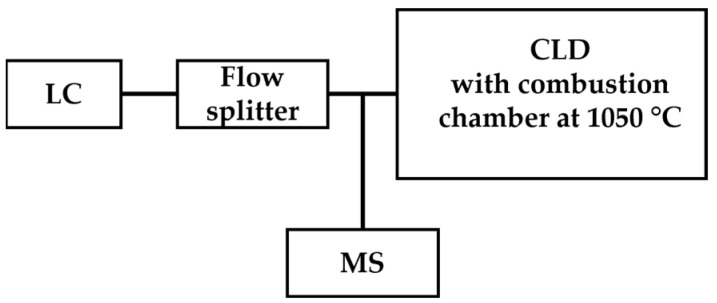
Schematic representation of LC-MS/CLD, as described in Ref. [67], featuring a flow splitter positioned after LC. The flow splitter divides eluents from LC into two streams, with one stream directed toward CLD for quantitative analysis and the other directed toward MS for qualitative analysis.

Mass spectrometry is a powerful detection technique based on the determination of the mass-to-charge ratio (m/z) of target ions and has been applied for the qualitative analysis and quantitation of ^•^NO-derived species such as nitrite and nitrate by coupling with chromatography as GC-MS or LC-MS [65,66]. Instead of sole MS detection, CLD was coupled for online quantitative analysis to LC-MS or GC-MS. For the sample class with a known formula, quantitation by CLD eliminates the need for preparing primary standards for each individual analyte within the known class with an unknown concentration, which allows high-throughput analysis. Hence, the combination of LC-MS/CLD shows the advantage of CLD for quantitation in high-throughput analysis and also brings the power of MS for specific demands of qualification. An example of this application is the analysis of 24 selected nitrogen-containing compounds such as C_10_H_10_N_2_, C_11_H_9_N_3_O_2_, etc. [67]. The scheme of LC-MS/CLD is shown in Figure 6.

The main pitfall of LC-MS/CLD techniques is the tailing of the peak, possibly due to clogging of the splitter and the nebulizer. This issue can be overcome with the adjustment of flow rate, tubing length, and replacement with a variable flow splitter [67]. LC-MS/CLD was applied for the identification and quantitation of in vivo metabolites in complex biological matrixes such as bile, urine, and plasma [68].

**Figure 7:**
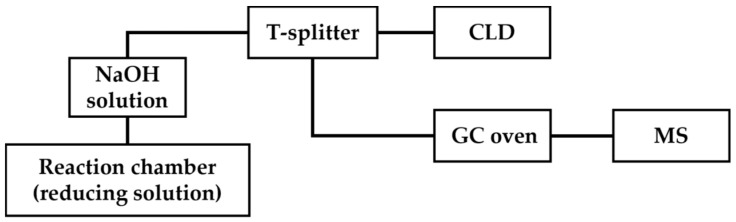
Schematic representation of GC-MS/CLD, as described in Ref. [69], featuring a T-splitter positioned after the NaOH solution chamber. The T-splitter divides the eluents into two streams, with one stream directed toward CLD for quantitative analysis and the other directed toward GC oven followed by MS detection.

In GC-MS/CLD, a T-splitter is placed after the trapping chamber containing a NaOH solution, which splits the ^•^NO generated by the chemiluminescence reaction into a chemiluminescence detector and mass spectrometer (Figure 7) [69]. With GC-MS/CLD, total ^•^NO can be quantified routinely with CLD; furthermore, the ^14^NO and isotope-labeled ^15^NO can be differentiated according to m/z with MS. An example application of this technique is the measurement of nitrite reductase activity in a macrophage cell line lysate. J774.2 macrophage cell lysate was treated with ^15^NO_3_^−^, which was then reduced to ^15^NO_2_^−^ by nitrate reductase activity. Both the original ^14^NO_2_^−^ and produced ^15^NO_2_^−^ were reduced with chemiluminescence assay to ^14^NO and ^15^NO, respectively, followed by MS analysis to distinguish them. In this case, GC-MS/CLD held the detection limit of ^•^NO-related products around 10 nM in 100 μL of the sample, which was demonstrated to be beneficial for the study of nitrate reductase activity or related enzymatic pathways [69].

### 4.2. Coupled with Microdialysis

Microdialysis is a technique used for sampling soluble molecules from the extracellular fluid through a semipermeable membrane. It is based on the principle of osmosis: molecules diffuse through a semipermeable membrane according to the concentration from an area of high concentration to an area of low concentration. It was found that by increasing the surface area of the dialysis membrane, the collection of samples from the interstitial space of the brain and other tissues became feasible [70]. A special microdialysis probe with a three-layer membrane was designed to collect ^•^NO in vivo from the blood or brain tissues of rats and rabbits, which was then combined with luminol-based chemiluminescence. This system was proposed for the real-time monitoring of changes in ^•^NO concentration in vivo, which allows to trace variations in ^•^NO metabolism in different physiological states, such as changes in body temperature. Microdialisis was also used to evaluate the impact of ^•^NO donors on the concentration of ^•^NO in blood and tissues [71].

### 4.3. Coupled with Flow Injection Analysis

**Figure 8:**
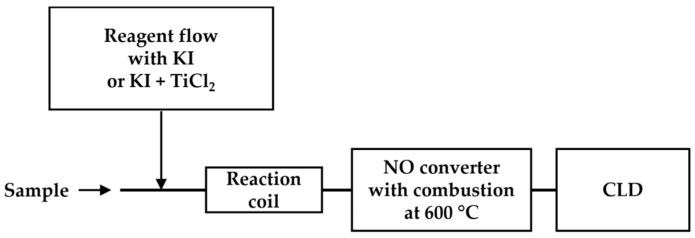
Schematic representation of FIA-CLD, as described in Ref. [73]. An aqueous sample is mixed with KI or the mixture (KI + TiCl_2_) before reaching the reaction coil. After high-temperature combustion in the ^•^NO converter, the ^•^NO products are detected using CLD.

Flow injection analysis (FIA) involves the injection of samples into a continuous carrier flow (a liquid stream), which then is mixed with other reagent flows for the reaction before reaching the detector [72,73,74]. Aqueous ammonia, nitrite, and nitrate were quantified using a flow injection system equipped with a chemiluminescence detector (Figure 8) [73,74]. In this system, an aqueous sample stream is continuously mixed with various reducing reagents (KI for the detection of nitrite only and titanium chloride for the detection of total nitrate and nitrite). Moreover, high-temperature combustion is applied to remove aqueous content. The detection limits of 10 nM for nitrite and nitrate were demonstrated in a performance study with standards.

Another FIA/chemiluminescence system was constructed for the monitoring of peroxynitrite in biological samples. This system is based on the collection of samples applying a dialysis membrane and the detection of peroxynitrite using luminol chemiluminescence [75]. With this setup, the detection limits were 10 and 100 pM for the calibrations with and without a membrane, respectively, which was proposed to be useful for in vivo monitoring of peroxynitrite as combined with microdialysis.

## 5. Advantages and Disadvantages of Chemiluminescence for Detecting ^•^NO Species in Biological Specimens

### 5.1. Advantages

Chemiluminescence is currently the most used technique for the quantification of ^•^NO in biological specimens [76] because of its high sensitivity, which allows for the detection of pM concentration of ^•^NO [77]; its selectivity over a wide dynamic linear range usually from 0.5 ppb to 5600 ppm ^•^NO [76]; its ability in the real-time monitoring of ^•^NO [78]; and its commercial availability with relatively affordable price.

As mentioned above, ozone-based chemiluminescence can be used not only for the detection of ^•^NO but also for nitrite, nitrate, S-nitrosothiols, nitrosyl–metal complex, and N-nitrosamines in biological samples [10,40,44,79,80,81,82,83].

### 5.2. Pitfalls

As mentioned above, an ozone-based chemiluminescence detector can detect the concentration of nitrite in the pM range. Therefore, it is important to avoid contamination coming from the water used for the preparation of reagents, standards, samples, and cleaning procedures. Milli-Q water was demonstrated to contain the lowest level of nitrite among the other sources of water and must be used for this application [84].

Contamination is not the only reason why sample preparation is a critical step. In fact, since ^•^NO has a short half-life and nitrite in the blood rapidly reacts with oxyhemoglobin to form nitrate, it is essential that the sample preparation is carried out in conditions that allow for the preservation of endogenous ^•^NO metabolites. This requires working quickly during both sample collecting and processing [85]. Moreover, different machines have different sensitivities. This variability arises from the fact that the reproducibility and sensitivity of the measurements are influenced by the ozone gas stream and the carrier gas flow rate, which are difficult to keep stable [86]. Therefore, for comparing data among experimental groups, the measurements need to be performed with the same machine [84].

Moreover, chemiluminescence methods require very specific equipment, time-consuming detection procedures, and frequent equipment maintenance, as well as a deep knowledge and understanding of the reactions that occur during measurement and data analysis [76,77]. Manual injection and manual data analysis (peak integration) are time-consuming and require experienced scientists.

However, if the proper precautions are taken to avoid contamination, interference in measurement, and errors in data analysis, ozone-based chemiluminescence can be considered one of the best techniques for the determination of ^•^NO metabolites, mainly due to its high sensitivity, high selectivity, and the possibility of coupling with other analytical techniques.

## 6. Alternatives for the Detection of ^•^NO Species in Biological Samples

CLD is one of the most widely used techniques for the determination of ^•^NO and ^•^NO derivatives, but many other methods are also currently in use. In this section, we give a quick overview of the alternatives that can be used, which are also summarized in Table 2.

### 6.1. Electrochemical Sensors (Electrodes)

An electrochemical sensor detects the electric current that is produced from the chemical reactions occurring at the electrode. The electrochemical detection of ^•^NO is based on the ability of ^•^NO to be oxidized or reduced. However, the oxidation of ^•^NO is mainly used in commercial electrodes [87,88]. There are also electrochemical sensors like the ion-selective electrode (ISE) that can be used specifically for nitrate detection (NO_3_^−^-ISE) [89].

### 6.2. Fluorescence

Fluorescent probes allow for the indirect detection of ^•^NO through its reaction with fluorophore, resulting in the formation of a fluorescent molecule. The most used fluorescent agents are 2,3-diaminonaphthalene (DAN) and diaminofluorescein (DAF). DANs react with nitrite under acidic conditions to produce fluorescent deprotonated naphthotriazol [90]. DAFs can be oxidized in the presence of ^•^NO, forming a fluorescent derivative triazolofluorescein [91,92]. The specificity and applicability of DANs and DAFs are limited by a number of issues, including the formation of secondary fluorescent products, the autocatalytic effects of the reactions, and the dependency of the reactions on the presence of other radicals and antioxidants.

### 6.3. Electron Paramagnetic Resonance (EPR)

EPR is a specific method used to indirectly detect ^•^NO formation in cells or tissues using spin traps like Hb, nitronylnitroxides, and the iron–dithiocarbamate complex [93,94,95]. Moreover, ^•^NO-derived produced in biological systems like nitrosylhemoglobin (HbNO) and DINCs can be specifically detected with EPR.

### 6.4. Membrane Inlet Mass Spectrometry

Membrane inlet mass spectrometry was used to measure ^•^NO in aqueous solution with a detection limit of 10 nM and linear response of 50 μM. This technique uses a semipermeable membrane that allows for the introduction of selectively small molecules like ^•^NO into the MS [96].

### 6.5. UV-Visible Spectrophotometry for ^•^NO Determination

Spectrometric detection of ^•^NO is based on the change in the absorption spectrum when oxyhemoglobin is oxidized to methemoglobin in the presence of ^•^NO in aqueous solution [97]. This method was applied to determine ^•^NO formation in endothelial cells. It has the advantage of being highly specific for ^•^NO. However, its applicability is limited to enzymatic reactions producing ^•^NO in the absence of background or interference.

### 6.6. Griess Assay

The Griess assay is a colorimetric method used for the determination of nitrite. This method is based on the formation of a red-violet azo compound (*λ*_max_ ≈ 540 nm) by the reaction of nitrite with the amino group of sulfanilic acid to form a diazonium cation, which then couples to α-naphthylamine. For the determination of nitrate, a reduction from nitrate to nitrite is needed before conducting the assay [98].

An automated system for the analysis of nitrite and nitrate was successfully established in 1982 [99]. Later, it was commercialized and developed as ENO-10, ENO-20, and the ENO-30 NO_x_ analyzer (Eicom Corporation) [100,101]. Such a system was constructed to obtain high sensitivity by coupling HPLC with the diazotization reaction method (Griess reaction). In brief, nitrite and nitrate in the samples are firstly separated on the reversed-phase HPLC column, followed by flowing through a copper-plated cadmium reduction column, where nitrate is reduced to nitrite. The resolved eluents then react with the Griess reagent to form the azo dye compounds, which are detected with a spectrophotometer [99,101].

An FIA/HPLC system was also applied for the reproducible determination of plasma nitrite levels based on the Griess reaction. This method may enable high-throughput measurements in plasma, e.g., clinical studies with a detection limit of 10 nM [102].

**Table 2:** Overview of the main techniques applied for the determination of ^•^NO and ^•^NO derivatives. The limit of detection, limit of quantitation, and dynamic range are highly dependent on the matrix and need to be determined for each experimental setting.

Method	Common Applications	Applications	Analytical Parameters	References
Electrochemical sensors	Direct on-line ^•^NO detection with electrooxidation or electroreduction	Real-time ^•^NO quantification in biological systems; ^•^NO detection in tissues and cells	Sensitivity: 3.5–106 pA per 1 µM change in ^•^NO concentration	[87,88]
Fluorescence	Indirect detection of ^•^NO with the formation of a fluorescent molecule	Detection of ^•^NO in cells and tissues	Limit of detection: 3–5 nM; Sensitivity: matrix-dependent	[90,91,92]
Electron paramagnetic resonance	Indirect detection of ^•^NO; direct detection of HbNO and dinitrosyl iron complex	^•^NO and HbNO detection in cells and tissues	Limit of detection: 500 nM	[93,94,95]
Mass spectrometry	Detection of ^•^NO with multiple ion detection	^•^NO detection in aqueous solution	Limit of detection: 10 nM	[96]
UV-visible spectrophotometry	Indirect detection of ^•^NO with oxyhemoglobin oxidation	^•^NO formation in cells and tissues	Limit of detection: 0.2 nmol/min	[97,103]
Griess assay	Determination of nitrite and nitrate with the formation of an azo dye in acidic condition	Determination of nitrite levels in biological systems	Limit of detection: 1–2 µM	[98,104]

## 7. Practical Considerations about Choosing CLD as Detection Method

Many techniques are capable of detecting ^•^NO species in biological samples. However, it is necessary to choose the technique depending on the specific needs of the experiments. For example, as the main players in the nitrate–nitrite-^•^NO/nitrosothiols biological pathway, nitrite and nitrate can be measured using the Griess assay (with the diazotiation of nitrite, where nitrate requires reduction to nitrite first) or using ozone-based CLD of ^•^NO [105,106,107,108]. The Griess assay with the colorimetric determination of the diazotization product is simple, fast, and affordable (chemicals and UV-vis spectrophotometer are relatively low costs as compared with other instruments). As mentioned before in Section 5, ozone-based chemiluminescence depends on the availability of a ^•^NO analyzer for the direct measurement of gaseous ^•^NO or those derived from the reduction of nitrite and nitrate. The detection procedures using ozone-based chemiluminescence also require more experience to avoid contamination and to carry out the reactions, but it is superior in sensitivity.

As reported by many researchers, the Griess assay has a limit of detection of around 1 μM in plasma [108,109,110,111]. In contrast, ozone-based chemiluminescence is able to quantify nitrite and other NO metabolites at the nM range in biological matrixes, which is much more sensitive compared with the Griess assay [43]. Moreover, direct quantification allows for an ozone-based chemiluminescence analyzer to measure even exhaled ^•^NO [112,113].

The choice of the technique needs to be based on the detection range and the sample type. For certain samples, such as wastewater or soil, with a rather complex matrix, a coupled technique such as HPLC-CLD or GC-CLD can be a solution instead of relying on CLD alone. As mentioned above, such a coupling system with chromatography provides additional separation before CLD, which reduces the matrix effect and the chance of under- or over-estimation. Furthermore, MS can bring highly accurate qualitative analysis and even the possibility of isotopic tracing with ^15^N-labeled substrate for the study of enzymatic pathways and metabolomics.

As with all semiquantitative and quantitative analytical techniques, calibrants should be prepared in a matrix as close as possible to the samples, which means the interference from other compounds in the sample matrix also needs to be included in the calibrants. The classes of target analytes (nitrite, nitrate, etc.) and their estimated levels in the samples need to be confirmed to define the calibration range. The detected concentrations of samples need to be located in the linear range of the calibration; otherwise, the samples need to be diluted.

Chemiluminescence depends on the reaction of ^•^NO with ozone. However, some species such as ethylenic hydrocarbons, sulfur compounds, and carbonyls can react with ozone and produce chemiluminescence signals, which may lead to interference for accurate detection [114]. Specifically, some substituted ethylenes can react with ozone and produce strong chemiluminescence signals at higher or lower pressure in the analyzer. Sulfur compounds, such as hydrogen sulfide or dimethyl sulfide, can produce intensive signals once they react with ozone. Carbonyls can be introduced from experimental treatments, such as treatment with CO to block oxy/deoxyhemoglobin, which interferes with detection by reacting with ozone. The disturbance of interferants like ethylenic hydrocarbons and sulfur to the ^•^NO/ozone reaction can be eliminated by increasing the detection wavelength to >600 nm (440 to 470 nm for ethylenic hydrocarbons, <400 nm for sulfur). However, even if some of the interference can be reduced or overcome with methods such as adjusting the wavelength, adjusting the pressure of the analyzer, or filtering, artificial contamination needs to be avoided or carefully evaluated before starting.

Some critical points for the technical procedures of ozone-based chemiluminescence in biological samples have been summarized in Table 3.

## 8. Successful Application in Different Fields

CLD has been applied to determine ^•^NO and its metabolites in different fields of research. The analysis of nitrogen species NO_x_ in the air for the monitoring of air pollution as well as the composition of exhausted air from motors and cars was one of the first applications of CLD for determining ^•^NO species and is still used today [115,116,117]. Another application is the determination of the concentration of NO_3_^−^ (derived from fertilizer) and other ^•^NO species in lakes, oceans, rivers, and drinking water [118,119,120].

The analysis of ^•^NO species in the gas phase is not only used for environmental monitoring but also for biomedical and clinical research. Already in 1991, it was shown that endogenous ^•^NO is present in the exhaled air of different animal species and humans and can be measured with CLD [121]. Follow-up studies found differences in exhaled ^•^NO in asthmatic patients compared with healthy individuals [122]. CLD was also applied to detect ^•^NO released by vasoactive drugs in exhaled air from pigs [123]. In this interesting pioneering study, the authors concluded that the measurement of exhaled ^•^NO could be a possible indicator of pulmonary endothelial dysfunction or hypertension. Measurement of exhaled ^•^NO is still used as a reproducible noninvasive indicator of inflammation of the airways [124,125].

The measurement of ^•^NO metabolites in plasma, urine, or tissues was also used to investigate the role of endogenous ^•^NO production or the effect of intake of nitrate in the body [126,127,128,129].

This is based on the principle that ^•^NO produced in the body is immediately oxidized into nitrite, nitrate, and nitroso compounds that can be measured as ^•^NO metabolites [130,131]. Nitrite concentration in plasma was proposed as an indicator of endothelial dysfunction and endothelial NO synthase (eNOS)-derived NO bioavailability [102,132]. Global eNOS knock-out (KO) mice show decreased circulating nitrite and nitrate metabolites [130,131,133,134]. Recently, we found that mice lacking eNOS in endothelial cells show a decrease in circulating nitrite and nitrate in plasma. However, restoring eNOS expression only in endothelial cells of global eNOS KO mice does not lead to the recovery of nitrite levels in the plasma. This indicates that the levels of nitrite in plasma are not only dependent on eNOS activity in the endothelium. Another finding of this cited study was that also eNOS expressed in RBCs contributes to the circulating nitrite levels [39,106].

In addition, the activity of nNOS and iNOS may also contribute to overall ^•^NO metabolites [131]. Recently, it was reported that COVID-19 patients show a decrease in ^•^NO and sulfide metabolites in plasma [135].

Due to the biological variability in circulating ^•^NO, nitrate, and nitrite levels, the measurements of these metabolites could not be used as clinical biomarkers until now. However, measuring these metabolites with CLD is still considered an important approach to understanding the systemic ^•^NO pathway in health and disease.

Regarding nitrate and nitrite levels in the circulation, it is important to note that a main factor in modulating those levels is the dietary intake of nitrate. There are multiple studies using CLD for analyzing nitrate and nitrite levels in food and drinking water [136], which is also important due to the fact that a high intake of nitrate has been considered harmful and cancerogenic due to the conversion of nitrate into nitrite by bacteria and further metabolism into nitrosamine [137,138]. Furthermore, sodium nitrate and potassium nitrate as well as sodium nitrite and potassium nitrite are used in the food industry, especially for the preservation of meat as additives [139]. Therefore, it was proposed to control nitrate and nitrite levels as a precaution to avoid potential health risks [140]. On the other hand, it has been shown that the supplementation of nitrate lowers blood pressure in humans, is cardioprotective, and shows the importance of dietary nitrate intake [128,129,141].

^•^NO signaling is known to play an important role in plant biology and is involved in multiple processes [142]. Similar to animals and humans, plants can use NADH and nitrite to generate ^•^NO but do not express a NO synthase. The measurement of these molecules is not only important for better insight into the signaling of plants but can also provide information about the accumulation of nitrate in the leaves and vegetables of plants due to the use of fertilizers.

In conclusion, the detection of ^•^NO with CLD is a widely used technique in multiple fields and is continuously improved and adapted for new possible usage.

## 9. Summary and Conclusions

The observation that ^•^NO reacts with ozone, leading to the emission of light, was first described and used to quantify ^•^NO by amplifying the signal in a photomultiplier tube. Chemiluminescence-based ^•^NO detection allowed researchers to confirm the nature of EDRF as ^•^NO and afterward to investigate ^•^NO and its derived species in biological samples in a specific, precise, and direct way.

Its high sensitivity and wide dynamic range make CLD an indispensable tool for quantitative analysis. To increase the applicability and specificity of the detection of nitrite, nitrate, or other metabolites, many scientists have combined the advantages of CLD with various analytical separative approaches, such as chromatography, MS, microdialysis, and FIA. These multilevel approaches enabled researchers to obtain both quantitative and qualitative profiles including the isotope tracing of ^14^NO and ^15^NO for enzymatic activity or in vivo monitoring of important ^•^NO-derived species such as nitrite and peroxynitrite.

Chemiluminescence-based ^•^NO detection has been applied in various fields, including the investigation of environmental pollution related to inorganic nitrates, as well as biomedical and clinical research on endogenous ^•^NO metabolites as indicators of endothelial dysfunction. It is important to note that ozone-based CLD is a highly sensitive method for the detection of ^•^NO species, but it needs to be operated under highly controlled conditions. Indeed, the system requires meticulous calibration, and both sample preparation and analysis must be conducted with careful consideration of sample composition and target analytes. Factors such as the presence of contaminants and molecules that could potentially interfere with the reactions in the chamber or the detector also need attention. Additionally, it is crucial to account for aspects like sample collection and storage, as well as technical issues such as system leakage, baseline fluctuations, contamination from surrounding air, and the exhaustion of ozone in the chamber.

In conclusion, chemiluminescence-based ^•^NO determination is a very useful and versatile tool for the quantitative analysis of ^•^NO and its metabolites in various samples and can be applied in many fields. The power of CLD for the detection of ^•^NO species can be further enhanced when coupled with other analytical separative techniques, delivering a more vivid picture of the status of ^•^NO metabolism in various samples. These techniques, coupled with the cell-specific genetic manipulation of NOS enzyme and/or pharmacological/dietetic intervention, will continue to reveal how ^•^NO metabolism is regulated in vivo.

## Figures and Tables

**Table 3:** Technical notes for performing ozone-based chemiluminescence in biological samples.

**1. Pretreatment**
*Stabilization reagent is prepared for organs, tissues, and cell lysates by adding* A total of 10 mM NEM, 2 mM ethylenediaminetetraacetic acid (EDTA) in PBS as lysis buffer. ○ NEM inhibits transnitrosation reactions by blocking SH groups and prevents artificial nitrosation. ○ EDTA chelates Fe2+ (and other metal ions) to avoid the Fenton reaction. In RBCs, ferricyanide is added by some authors to oxidize hemoglobin and block hemoglobin-dependent NO^2−^ oxidation or reduction.
*Differentiation assistant reagent: measurements in tri-iodide reductive solution in…* Direct injection = NO_2_^−^ + total nitroso species. Addition of 10% sulfanilamide in 1 M HCl = total nitroso species (converting nitrite to a diazonium cation, which is not detectable). Addition of HgCl_2_ then 10% sulfanilamide = mercury-resistant nitroso compounds.
**2. Avoid contamination**
*Nitrite contamination is everywhere; therefore, pay attention to…* Check buffers and Milli-Q water for nitrite contamination before use. ○ NEM, EDTA solution for stabilization during blood collection. ○ Lysis buffer containing NEM, EDTA in PBS. ○ Milli-Q water for dissolving nitrite standard and diluted calibrants. Clean plastic and glassware thoroughly by washing with Milli-Q water before use. ○ For storing organs, blood, or RBC and plasma. ○ For preparing lysis buffer and homogenizing tissues. ○ Rinsing syringe with water and alcohol between each injection. Evaluate the contamination of NEM and EDTA, which can be quite contaminated with nitrite and varied in different lot numbers.
**3. Minimum time before storage**
*The scavenging of nitrite can be really fast; therefore,* Collect and weigh organs fast during collection (recording the weight further normalization) (< 2 to 3 min). Keep the collection and centrifugation of blood (to separate plasma and RBCs) as short and constant as possible (centrifugation at 4000× *g* for 3 min for mice blood or 800× *g* for 10 min for human samples).
**4. Sample storage**
Snap-freeze all the samples including organ tissues, RBC, and plasma in liquid nitrogen immediately after weighing or centrifugation. Store the frozen samples at −80 °C until use. Avoid freeze–thawing.
**5. Measurement procedure**
I. Prepare the reductive/oxidative solution. Reductive solution: 0.405 g of KI, 0.143 g of I_2_, 3.75 mL Milli-Q water, and 50 mL of glacial acetic acid. Oxidative solution: 1.646 g potassium ferricyanide of in 100 mL PBS, pH 7.5. II. Add the reductive/oxidative solution to the reaction chamber. Maintain the purging flow of nitrogen constant. Keep the water bath temperature of the chambers constant (60 °C for the reductive reaction and 37 °C for the oxidative reaction). Wait until the baseline becomes steady. III. Inject the standards/samples into the reaction chamber. Inject fast to the reaction chamber. Avoid air entering into the chamber, which may lead to artifacts and contaminants. Pay attention to the signals on the display immediately after injection. Disconnect the connector between the reaction chamber and the chemiluminescence detector once significant signals are observed from contamination to avoid chemiluminescence detector being saturated.
**6. Technical issues**
Check for any leakage from reaction chamber (it must be fully sealed) or the connection tubings. ○ Leakage might lead to changes in baseline and affect the accuracy of the detection and the sensitivity (signal-to-noise ratio).
**7. pH**
A stable pH of the reductive solution (normally pH around 2.5) is critical to control the interferences of side reactions. For the oxidative solution with potassium ferricyanide, the pH is neutral.

## Data Availability

Not applicable.
